# How to do an enucleation for retinoblastoma

**Published:** 2018-06-03

**Authors:** Swathi Kaliki

**Affiliations:** 1 Head of The Operation Eyesight Universal Institute for Eye Cancer: LV Prasad Eye Institute, Hyderabad, India.

**Despite many advances in treatment, removal of the eye is sometimes unavoidable and can bring resistance from parents. Safe enucleation with implant followed by a well fitted prosthesis will encourage other parents to agree to this life-saving procedure.**

**Figure:**
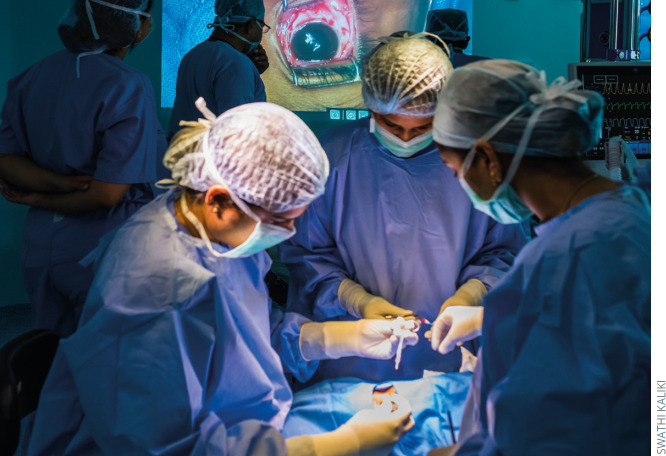
The eye is prepared for surgery. INDIA

The three main goals of treatment of retinoblastoma are to save the child's life, to keep the eye, and to preserve vision. With recent advances in the management of retinoblastoma, especially with the introduction of chemotherapy, the need for enucleation has significantly reduced. However, enucleation is still the treatment of choice in cases with advanced intraocular retinoblastoma or in cases where saving the globe has failed.

Though the basic principles of surgery remain the same^1^, a recent survey of 58 surgeons in 32 countries on enucleation techniques and implants in retinoblastoma revealed wide variations in practice.^2^ In this article, we will discuss the surgical steps of enucleation and implant placement using the myoconjunctival technique for retinoblastoma.

## Indications for enucleation

**Primary enucleation** is the preferred treatment in eyes with advanced unilateral intraocular retinoblastoma, corresponding to Group E in the International Intraocular Retinoblastoma Classification (Table 1, p. 11).

Secondary enucleation is performed in:

- Eyes that have failed conservative treatment strategies
- Phthisical eyes after-high-dose chemotherapy (see article on treatment of extraocular Rb).

## Pre-operative evaluation

Prior to performing an enucleation for retinoblastoma, it is important to try and exclude metastatic disease. Bone marrow evaluation and cerebrospinal fluid analysis is recommended in cases with advanced retinoblastoma. If possible, orbital imaging by computerised tomography (CT) or magnetic resonance imaging (MRI) should be performed prior to enucleation to rule out extrascleral tumour extension or gross optic nerve involvement, which is seen as optic nerve thickening on the scan.

In cases with gross optic nerve thickening or those with extrascleral extension, systemic chemotherapy is recommended as the primary treatment, and after regression of the extraocular tumour, enucleation is performed as secondary treatment.

Since the operation is performed under general anaesthesia, suitable investigations must also be performed. Blood haemoglobin levels of a minimum of 10 grams per decilitre, white blood cell count of <15,000 per cubic millimetre, and a platelet count of >100,000 per cubic millimetre of blood are preferred.

## Myoconjunctival technique of enucleation and implant

There should be minimal manipulation of the globe during the operation. Avoid perforating the globe and obtain an adequate length of optic nerve (>15 mm).

Here we describe the surgical technique practiced in our centre, which ensures minimal globe manipulation and produces an adequate length of optic nerve. Go to **www.dropbox.com/s/7gbnovs9lyeokoz/N302626%20enucleation.mp4?dl=0** to see a video for the surgical steps of the procedure.

1. Perform indirect ophthalmoscopy before starting the operation to confirm the eye procedure will be done on the correct eye.
2. Gently place a wire speculum.
3. If the globe is enlarged or the orbit is small or tight, perform a lateral canthotomy to increase the working space.
4. Use conjunctival scissors to perform a perilimbal conjunctival peritomy around the whole eye. Take care to preserve the conjunctiva - handle it gently to minimise post-operative conjunctival scarring.
5. Perform a tenotomy in all four quadrants using curved tenotomy scissors. The dissection should be carried out to the equator of the globe in order to ease prolapse of the globe in the later stages of surgery.
6. Gently place a muscle hook under each of the four rectus muscles. Place muscle traction sutures 2 to 3 mm from the muscle insertion. Be gentle during needle entry into the muscle to avoid globe perforation.
7. The order of tagging and cutting the rectus muscles is based on the distance to the limbus: first medial, then inferior, then lateral, and finally the superior rectus. Insert absorbable muscle-tagging sutures through the muscle, 4 to 5 mm from the traction sutures. Cut the muscles in between the traction suture and tag suture with conjunctival scissors.
8. The superior oblique and inferior oblique muscles are now identified and cut. It is preferable to use cautery to cut these muscles in order to minimise bleeding during surgery.
9. After all six of the extraocular muscles have been severed, use the four traction sutures to exert gentle traction on the globe and facilitate globe prolapse. This is an important step to obtain an adequate length of optic nerve subsequently. If there is resistance to globe prolapse, there may be several reasons. If the eye speculum is too tight, replace it with the correct eye speculum. If the surgical space is too narrow (due to a small orbit), perform a small lateral canthotomy or a relaxing horizontal conjunctival incision laterally. It may also be due to incomplete severing of extraocular muscles, so check these again.
10. Curved tenotomy scissors are then inserted by the lateral approach and the optic nerve is identified near the orbital apex.
11. The optic nerve is then cut just above the orbital apex to avoid damage to the important structures there. This ensures adequate length of the optic nerve (>15 mm). Give hypotensive anesthesia and/or a reverse Trendelenburg position (the head is 15–30 degrees higher than the feet) to ensure minimal bleeding during this step.
12. Pack the socket immediately with gauze and keep in place for 5 minutes to stop bleeding and avoid the formation of a haematoma.
13. Inspect the enucleated globe for any evidence of extrascleral extension of the tumour. Measure the length of optic nerve using calipers.
14. Send the globe for detailed histopathology analysis.
15. After stopping the bleeding, identify the posterior Tenon's capsule. Place an adequate-sized implant in the intraconal space. The implant is secured in place by suturing the posterior Tenon's capsule with absorbable sutures. The author prefers to use non-integrated implant for retinoblastoma cases.
16. The tag sutures attached to the cut end of the recti muscles are then brought out externally through the conjunctival fornices.
17. The anterior Tenon's capsule and the conjunctiva are then closed with absorbable sutures in two layers.
18. The tag sutures are then knotted to each other, thus completing the myoconjunctival technique of enucleation.
19. An iris-painted or a plain conformer with a draining pore is then placed in the socket. The conformer can be secured in place with central suture tarsorrhaphy.
20. A pressure patch is applied for 24 hours.

**Figure 1 (A-U):**
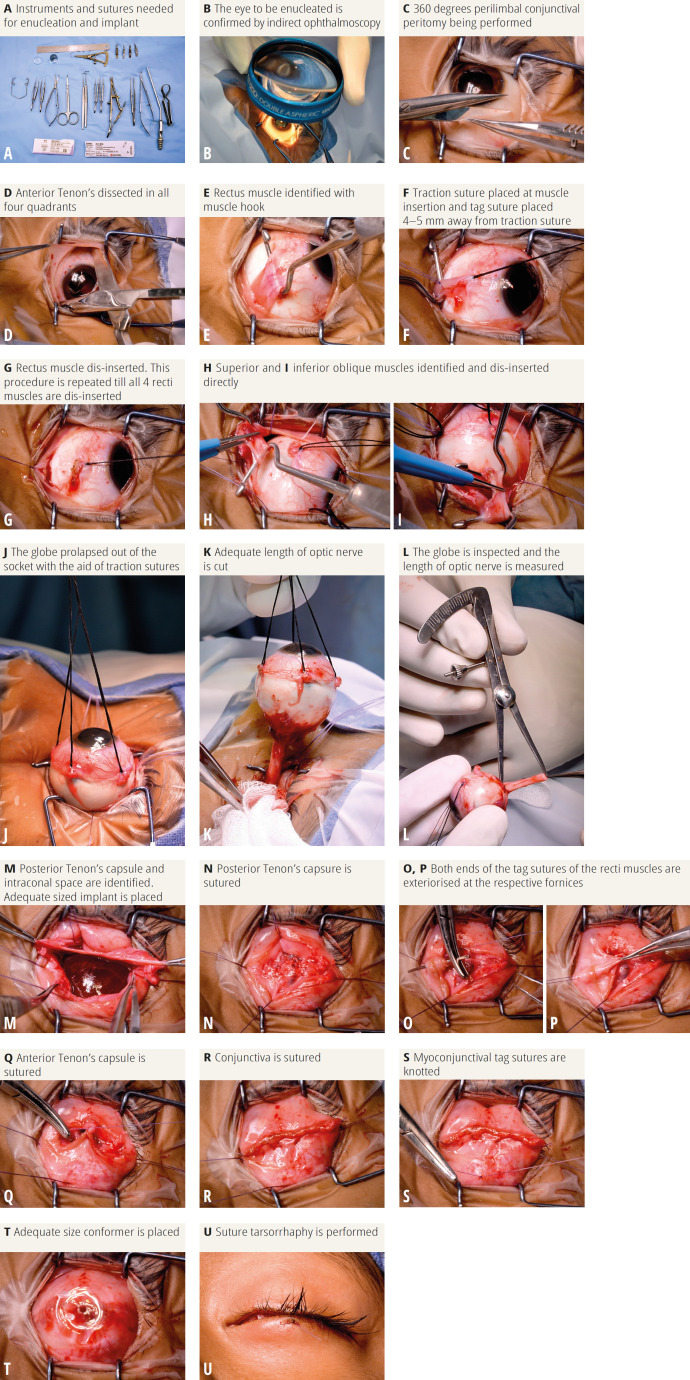
Surgical steps of enucleation and implant for retinoblastoma

## Postoperative care

- The pressure patch is removed after 24 hours
- Patient is given oral antibiotics for 1 week
- Topical antibiotics are prescribed for 2 weeks and topical steroids are tapered over 6 weeks
- The suture tarsorrhaphy is removed after 1 week
- Based on the histopathology report, further treatment may be required. Presence of high-risk features on histopathology (refer to page 17 for further details) are an indication for adjuvant systemic chemotherapy
- Dispense customised ocular prosthesis 6 weeks after enucleation.
